# Tribonacci numbers that are concatenations of two repdigits

**DOI:** 10.1007/s13398-020-00933-0

**Published:** 2020-09-15

**Authors:** Mahadi Ddamulira

**Affiliations:** Institute of Analysis and Number Theory, University of Technology, Kopernikusgasse 24/II, 8010 Graz, Austria

**Keywords:** Tribonacci number, repdigit, linear form in logarithms, reduction method, Primary 11B39, 11D45, Secondary 11D61, 11J86

## Abstract

Let $$ (T_{n})_{n\ge 0} $$ be the sequence of Tribonacci numbers defined by $$ T_0=0 $$, $$ T_1=T_2=1$$, and $$ T_{n+3}= T_{n+2}+T_{n+1} +T_n$$ for all $$ n\ge 0 $$. In this note, we use of lower bounds for linear forms in logarithms of algebraic numbers and the Baker-Davenport reduction procedure to find all Tribonacci numbers that are concatenations of two repdigits.

## Introduction

A *repdigit* is a positive integer *R* that has only one distinct digit when written in its decimal expansion. That is, *R* is of the form

1.1 $$\begin{aligned} R=\overline{\underbrace{d\cdots d}_{\ell {\text {times}}}}=d\left( \dfrac{10^{\ell }-1}{9}\right) , \end{aligned}$$

for some positive integers $d, \ell$ with $\ell \ge 1$ and $0\le d\le 9$. The sequence of repdigits is sequence *A*010785 on the On-Line Encyclopedia of Integer Sequences (OEIS) [8].

Consider the sequence $(T_{n})_{n\ge 0}$ of *Tribonacci* numbers given by

$$\begin{aligned} T_0=0, \quad T_1= 1, \quad T_2=1, \quad \hbox {and} \quad T_{n+3}= T_{n+2}+T_{n+1}+T_n \quad \hbox {for all}\quad n\ge 0. \end{aligned}$$

The sequence of Tribonacci numbers is sequence *A*000073 on the OEIS. The first few terms of this sequence are given by

$$\begin{aligned} (T_{n})_{n\ge 0} = \{0, 1, 1, 2, 4, 7, 13, 24, 44, 81, 149, 274, 504, 927, 1705, 3136, \cdots \}. \end{aligned}$$

## Main result

In this paper, we study the problem of finding all Tribonacci numbers that are concatenations of two repdigits. More precisely, we completely solve the Diophantine equation

2.1 $$\begin{aligned} T_{n} = \overline{{\underbrace{d_1 \cdots d_1}_{\ell _1\,{\text {times}}}} {\underbrace{d_2 \cdots d_2}_{\ell _2 \,{\text {times}}}}} = d_1 \left( \dfrac{10^{\ell _q}-1}{9}\right) \cdot 10^{\ell _2}+d_2\left( \dfrac{10^{\ell _2}-1}{9}\right) , \end{aligned}$$

in non-negative integers $(n, d_1, d_2, \ell _1, \ell _2)$ with $n \ge 0$, $\ell _1 \ge \ell _2 \ge 1$, and $d_1, d_2 \in \{0, 1, \ldots , 9\}, d_1>0$.

Our main result is the following.

### Theorem 2.1

The only Tribonacci numbers that are concatenations of two repdigits are

$$\begin{aligned} T_n \in \{13,24,44,81\}. \end{aligned}$$

Our method of proof involves the application of Baker’s theory for linear forms in logarithms of algebraic numbers, and the Baker-Davenport reduction procedure. Computations are done with the help of a computer program in Mathematica.

Let $$ (F_{n})_{n\ge 0} $$ be the sequence of *Fibonacci* numbers given by $$ F_0=0, ~ F_1= 1,$$ and $$ F_{n+2}= F_{n+1}+F_{n} $$ for all $$ n\ge 0 $$, and $$ (B_{n})_{n\ge 0} $$ be the sequence of *balancing* numbers given by $$ B_0=0, ~ B_1= 1,$$ and $$ B_{n+2}= 6B_{n+1}-B_{n} $$ for all $$ n\ge 0 $$. This paper is inspired by the results of Alahmadi et al. [1], in which they show that the only Fibonacci numbers that are concatenations of two repdigits are $$F_n \in \{13,21,34,55,89,144,233,377\}$$, and Rayaguru and Panda [10], who showed that $$ B_n\in \{35\} $$ is the only balancing number that can be written as a concatenation of two repdigits. Other related interesting results in this direction include: the result of Bravo and Luca [3], the result of Trojovský [11], the result of Qu and Zeng [9], and the result of Boussayoud et al. [2].

## Preliminary results

### The Tribonacci sequence

Here, we recall some important properties of the Tribonacci sequence $\{T_n\}_{n\ge 0}$. The characteristic equation

$$\begin{aligned} \Psi (x):=x^3-x^2-x-1 = 0, \end{aligned}$$

has roots $\alpha , \beta , \gamma = \bar{\beta }$, where

3.1 $$\begin{aligned} \alpha =\dfrac{1+(r_1+r_2)}{3}, \qquad \beta = \dfrac{2-(r_1+r_2)+\sqrt{-3}(r_1-r_2)}{6}, \end{aligned}$$

and

3.2 $$\begin{aligned} r_1=\root 3 \of {19+3\sqrt{33}} \quad \hbox {and}\quad r_2=\root 3 \of {19-3\sqrt{33}}. \end{aligned}$$

Further, the Binet formula for the general terms of the Tribonacci sequence is given by

3.3 $$\begin{aligned} T_n = a\alpha ^{n}+b\beta ^{n}+c\gamma ^{n} \qquad \hbox { for all} \quad n\ge 0, \end{aligned}$$

where

3.4 $$\begin{aligned} \quad a=\dfrac{1}{(\alpha -\beta )(\alpha -\gamma )}, \quad b= \dfrac{1}{(\beta -\alpha )(\beta -\gamma )}, \quad c = \dfrac{1}{(\gamma -\alpha )(\gamma -\beta )}=\bar{b}. \end{aligned}$$

Furthermore,

$$\begin{aligned} a=\dfrac{\alpha }{\alpha ^{2}+2\alpha +3}, \end{aligned}$$

and the minimal polynomial of *a* over the integers is given by

$$\begin{aligned} 44x^3+4x-1, \end{aligned}$$

has zeros *a*,  *b*,  *c* with $\max \{|a|, ~|b|, ~|c|\} < 1$. Numerically, the following estimates hold:

3.5 $$\begin{aligned} 1.83< & {} \alpha<1.84;\nonumber \\ 0.73< |\beta |= & {} |\gamma |=\alpha ^{-\frac{1}{2}}< 0.74;\nonumber \\ 0.18< & {} a<0.19;\nonumber \\ 0.35< & {} |b|=|c|<0.36. \end{aligned}$$

From (3.1), (3.2), and (3.5), it is easy to see that the contribution the complex conjugate roots $\beta$ and $\gamma$, to the right-hand side of (3.3), is very small. In particular, setting

3.6 $$\begin{aligned} e(n):=T_n-a\alpha ^{n}=b\beta ^{n}+c\gamma ^{n}\quad \hbox { then } \quad |e(n)|< \dfrac{1}{\alpha ^{n/2}}, \end{aligned}$$

holds for all $n\ge 1$. The proof of the last inequality in (3.6) follows from the fact that $|\beta |=|\gamma |=\alpha ^{-\frac{1}{2}}$ and $|b|=|c|< 0.36$ (by 3.5). That is, for any $n\ge 1$,

$$\begin{aligned} |e(n)|=\left| b\beta ^{n}+c\gamma ^{n}\right| \le |b||\beta |^{n}+ |c||\gamma |^{n}= |b|\alpha ^{-\frac{n}{2}}+ |c| \alpha ^{-\frac{n}{2}}< 2\cdot 0.36 \cdot \alpha ^{-\frac{n}{2}} < \dfrac{1}{\alpha ^{n/2}}. \end{aligned}$$

Furthermore, by induction, one can prove that

3.7 $$\begin{aligned} \alpha ^{n-2}\le T_n \le \alpha ^{n-1} \quad \hbox {holds for all }\quad n\ge 1. \end{aligned}$$

Let ${\mathbb {K}}:={\mathbb {Q}}(\alpha , \beta )$ be the splitting field of the polynomial $\Psi$ over ${\mathbb {Q}}$. Then, $[{\mathbb {K}}, {\mathbb {Q}}]=6$. Furthermore, $[{\mathbb {Q}}(\alpha ):{\mathbb {Q}}]=3$. The Galois group of ${\mathbb {K}}$ over ${\mathbb {Q}}$ is given by

$$\begin{aligned} {\mathcal {G}}:=\hbox {Gal}({\mathbb {K/Q}})\cong \{(1), (\alpha \beta ),(\alpha \gamma ), (\beta \gamma ), (\alpha \beta \gamma ), (\alpha \gamma \beta )\} \cong S_3. \end{aligned}$$

Thus, we identify the automorphisms of ${\mathcal {G}}$ with the permutations of the zeros of the polynomial $\Psi$. For example, the permutation $(\alpha \gamma )$ corresponds to the automorphism $\sigma : \alpha \rightarrow \gamma , ~\gamma \rightarrow \alpha , ~\beta \rightarrow \beta$.

### Linear forms in logarithms

Let $\eta$ be an algebraic number of degree *d* with minimal primitive polynomial over the integers

$$\begin{aligned} a_{0}x^{d}+ a_{1}x^{d-1}+\cdots +a_{d} = a_{0}\prod _{i=1}^{d}(x-\eta ^{(i)}), \end{aligned}$$

where the leading coefficient $a_{0}$ is positive and the $\eta ^{(i)}$’s are the conjugates of $\eta$. Then the logarithmic height of $\eta$ is given by

$$\begin{aligned} h(\eta ) := \dfrac{1}{d}\left( \log a_{0} + \sum _{i=1}^{d}\log \left( \max \{|\eta ^{(i)}|, 1\}\right) \right) . \end{aligned}$$

In particular, if $\eta = p/q$ is a rational number with $\gcd (p,q) = 1$ and $q>0$, then $h(\eta ) = \log \max \{|p|, q\}$. The following are some of the properties of the logarithmic height function $h(\cdot )$, which will be used in the next section of this paper without reference:

$$\begin{aligned} h(\eta _1\pm \eta _2)&\le h(\eta _1) +h(\eta _2) +\log 2;\\ h(\eta _1\eta _2^{\pm 1})&\le h(\eta _1) + h(\eta _2);\\ h(\eta ^{s})&= |s|h(\eta ) \quad (s\in {\mathbb {Z}}). \end{aligned}$$

We recall the result of Bugeaud et al. ([4], Theorem 9.4), which is a modified version of the result of Matveev [7], which is one of our main tools in this paper.

#### Theorem 3.1

Let $\eta _1,\cdots ,\eta _t$ be positive real algebraic numbers in a real algebraic number field ${\mathbb {K}} \subset {\mathbb {R}}$ of degree *D*, $b_1,\cdots ,b_t$ be nonzero integers, and assume that

$$\begin{aligned} \Lambda :=\eta _1^{b_1}\cdots \eta _t^{b_t} - 1\ne 0. \end{aligned}$$

Then,

$$\begin{aligned} \log |\Lambda | > -1.4\cdot 30^{t+3}\cdot t^{4.5}\cdot D^{2}(1+\log D)(1+\log B)A_1\cdots A_t, \end{aligned}$$

where

$$\begin{aligned} B\ge \max \{|b_1|, \cdots , |b_t|\}, \end{aligned}$$

and

$$\begin{aligned} A_i \ge \max \{Dh(\eta _i), |\log \eta _i|, 0.16\},\qquad {\hbox {for all}}\qquad i=1,\cdots ,t. \end{aligned}$$

### Reduction procedure

During the calculations, we get upper bounds on our variables which are too large, thus we need to reduce them. To do so, we use some result from the theory of continued fractions. For a nonhomogeneous linear form in two integer variables, we use a slight variation of a result due to Dujella and Pethő ([5], Lemma 5a). For a real number *X*, we write $\Vert X\Vert := \min \{|X-n|: n\in {\mathbb {Z}}\}$ for the distance from *X* to the nearest integer.

#### Lemma 3.1

Let *M* be a positive integer, $\frac{p}{q}$ be a convergent of the continued fraction expansion of the irrational number $\tau$ such that $q>6M$, and $A,B,\mu$ be some real numbers with $A>0$ and $B>1$. Furthermore, let $\varepsilon : = \Vert \mu q\Vert -M\Vert \tau q\Vert$. If $\varepsilon > 0$, then there is no solution to the inequality

$$\begin{aligned} 0<|u\tau -v+\mu |<AB^{-w}, \end{aligned}$$

in positive integers *u*, *v*, and *w* with

$$\begin{aligned} u\le M \quad {\hbox {and}}\quad w\ge \dfrac{\log (Aq/\varepsilon )}{\log B}. \end{aligned}$$

The following Lemma is also useful. It is due to Gúzman Sánchez and Luca ([6], Lemma 7).

#### Lemma 3.2

If $r\ge 1$, $H>(4r^2)^r$, and $H>L/(\log L)^r$, then

$$\begin{aligned} L<2^rH(\log H)^r. \end{aligned}$$

## The proof of Theorem 2.1

### The small ranges

With the help of Mathematica, we checked all the solutions to the Diophantine equation (2.1) in the ranges $0\le d_2< d_1\le 9$ and $1\le \ell _2 \le \ell _1 \le n \le 200$ and found only the solutions stated in Theorem 2.1. From now on we assume that $n>200$.

### The initial bound on *n*

We rewrite (2.1) as

$$\begin{aligned} T_n= & {} \overline{\underbrace{d_1\cdots d_1}_{\ell _1 \,\hbox { times}} \underbrace{d_2\cdots d_2}_{\ell _2 \,\hbox { times}}}\\= & {} \overline{\underbrace{d_1\cdots d_1}_{\ell _1 \,\hbox { times}}} \cdot 10^{\ell _2}+ \overline{ \underbrace{d_2\cdots d_2}_{\ell _2 \,\hbox { times}}}\\= & {} d_1\left( \dfrac{10^{\ell _1}-1}{9}\right) \cdot 10^{\ell _2}+d_2 \left( \dfrac{10^{\ell _2}-1}{9}\right) \quad (\hbox {by} \quad (1.1))\\= & {} \dfrac{1}{9}\left( d_1\cdot 10^{\ell _1+\ell _2}-(d_1-d_2)\cdot 10^{\ell _2} - d_2\right) . \end{aligned}$$

Thus,

4.1 $$\begin{aligned} T_n=\dfrac{1}{9}\left( d_1\cdot 10^{\ell _1+\ell _2}-(d_1-d_2)\cdot 10^{\ell _2} - d_2\right) . \end{aligned}$$

We prove the following lemma, which gives a relation on the size of *n* versus $\ell _1+\ell _2$.

#### Lemma 4.1

All solutions of the Diophantine equation (4.1) satisfy

$$\begin{aligned} (\ell _1+\ell _2)\log 10 -2< n\log \alpha < (\ell _1+\ell _2)\log 10 +2. \end{aligned}$$

#### Proof

The proof follows easily from (3.7). One can see that

$$\begin{aligned} \alpha ^{n-2}\le T_n < 10^{\ell _1+\ell _2}. \end{aligned}$$

Taking the logarithm on both sides, we get that

$$\begin{aligned} (n-2)\log \alpha < (\ell _1+\ell _2)\log 10, \end{aligned}$$

which leads to

4.2 $$\begin{aligned} n\log \alpha< (\ell _1+\ell _2)\log 10+2 \log \alpha < (\ell _1+\ell _2)\log 10 +2. \end{aligned}$$

For the lower bound, we have that

$$\begin{aligned} 10^{\ell _1+\ell _2-1}<T_n\le \alpha ^{n-1}. \end{aligned}$$

Taking the logarithm on both sides, we get that

$$\begin{aligned} (\ell _1+\ell _2-1)\log 10 < (n-1)\log \alpha , \end{aligned}$$

which leads to

4.3 $$\begin{aligned} (\ell _1+\ell _2)\log 10-2< (\ell _1+\ell _2-1)\log 10+\log \alpha < n\log \alpha . \end{aligned}$$

Comparing (4.2) and (4.3) gives the result in the lemma. $\square$

Next, we examine (4.1) in two different steps.

**Step 1** Substituting (3.3) in (4.1), we get that

$$\begin{aligned} a\alpha ^{n}+b\beta ^{n}+c\gamma ^{n}=\dfrac{1}{9}\left( d_1\cdot 10^{\ell _1+\ell _2}-(d_1-d_2)\cdot 10^{\ell _2} - d_2\right) . \end{aligned}$$

By (3.6), this is equivalent to

$$\begin{aligned} 9a\alpha ^{n}-d_1\cdot 10^{\ell _1+\ell _2}=-9e(n)-(d_1-d_2)\cdot 10^{\ell _2}-d_2, \end{aligned}$$

from which we deduce that

$$\begin{aligned} \left| 9a\alpha ^{n}-d_1\cdot 10^{\ell _1+\ell _2}\right|= & {} \left| 9e(n)+(d_1-d_2) \cdot 10^{\ell _2}+d_2\right| \\\le & {} 9\alpha ^{-n/2}+9\cdot 10^{\ell _2}+9\\< & {} 28\cdot 10^{\ell _2}. \end{aligned}$$

Thus, dividing both sides by $d_1\cdot 10^{\ell _1+\ell _2}$ we get that

4.4 $$\begin{aligned} \left| \left( \dfrac{9a}{d_1}\right) \cdot \alpha ^{n}\cdot 10^{-\ell _1-\ell _2} -1\right|< \dfrac{28\cdot 10^{\ell _2}}{d_1\cdot 10^{\ell _1+\ell _2}}< \dfrac{28}{10^{\ell _1}}. \end{aligned}$$

Put

4.5 $$\begin{aligned} \Lambda _1:=\left( \dfrac{9a}{d_1}\right) \cdot \alpha ^{n}\cdot 10^{-\ell _1-\ell _2}-1. \end{aligned}$$

Next, we apply Theorem 3.1 on (4.5). First, we need to check that $\Lambda _1 \ne 0$. If it were, then we would get that

$$\begin{aligned} a\alpha ^{n}=\dfrac{d_1}{9}\cdot 10^{\ell _1+\ell _2}. \end{aligned}$$

Now, we apply the automorphism $\sigma$ of the Galois group ${\mathcal {G}}$ on both sides and take absolute values as follows.

$$\begin{aligned} \left| \dfrac{d_1}{9}\cdot 10^{\ell _1+\ell _2}\right| =\left| \sigma (a\alpha ^{n})\right| = \left| c\gamma ^{n}\right| <1, \end{aligned}$$

which is false. Thus, $\Lambda _1\ne 0$. So, we apply Theorem 3.1 on (4.5) with the data:

$$\begin{aligned} t:=3, \quad \eta _1:=\dfrac{9a}{d_1}, \quad \eta _2:=\alpha , \quad \eta _3:=10, \quad b_1:=1, \quad b_2:=n, \quad b_3:=-\ell _1-\ell _2. \end{aligned}$$

By Lemma 4.1, we have that $\ell _1+\ell _2 < n$. Therefore, we can take $B:=n$. Observe that ${\mathbb {K}}:={\mathbb {Q}}(\eta _1,\eta _2, \eta _3)={\mathbb {Q}}(\alpha )$, since $a=\alpha /(\alpha ^2 +2\alpha +3)$, so $D:=3$. We have

$$\begin{aligned} h(\eta _1)=h(9a/d_1)\le h(9)+h(a)+h(d_1)\le \log 9+\frac{1}{3}\log 44 +\log 9 \le 5.66. \end{aligned}$$

Furthermore, $h(\eta _2)=h(\alpha )=(1/3)\log \alpha$ and $h(\eta _3)=h(10)=\log 10$. Thus, we can take

$$\begin{aligned} A_1:=16.98, \quad A_2:=\log \alpha , \quad \hbox {and} \quad A_3:=3\log 10. \end{aligned}$$

Theorem 3.1 tells us that

$$\begin{aligned} \log |\Lambda _1|> & {} -1.4\cdot 30^{6}\cdot 3^{4.5}\cdot 3^{2}(1+\log 3) (1+\log n)(16.98)(\log \alpha )(3\log 10)\\> & {} -1.94\cdot 10^{14}(1+\log n). \end{aligned}$$

Comparing the above inequality with (4.4) gives

$$\begin{aligned} \ell _1\log 10-\log 28 < 1.94\cdot 10^{14}(1+\log n), \end{aligned}$$

leading to

4.6 $$\begin{aligned} \ell _1\log 10 < 1.96\cdot 10^{14}(1+\log n). \end{aligned}$$

**Step 2** By (3.6), we rewrite (4.1) as

$$\begin{aligned} 9a\alpha ^{n}-\left( d_1\cdot 10^{\ell _1}-(d_1-d_2)\right) \cdot 10^{\ell _2}=-9e(n)-d_2, \end{aligned}$$

from which we deduce that

$$\begin{aligned} \left| 9a\alpha ^{n}-\left( d_1\cdot 10^{\ell _1}-(d_1-d_2)\right) \cdot 10^{\ell _2}\right| =\left| 9e(n)+d_2\right| \le 9\alpha ^{-n/2}+9 < 18. \end{aligned}$$

Thus, dividing both sides by $9a\alpha ^{n}$ we get that

4.7 $$\begin{aligned} \left| \left( \dfrac{d_1\cdot 10^{\ell _1}-(d_1-d_2)}{9a}\right) \cdot \alpha ^{-n}\cdot 10^{\ell _2} -1\right|< \dfrac{18}{9a\alpha ^{n}}< \dfrac{2}{\alpha ^{n}}. \end{aligned}$$

Put

4.8 $$\begin{aligned} \Lambda _2:=\left( \dfrac{d_1\cdot 10^{\ell _1}-(d_1-d_2)}{9a}\right) \cdot \alpha ^{-n}\cdot 10^{\ell _2} -1. \end{aligned}$$

Next, we apply Theorem 3.1 on (4.8). First, we need to check that $\Lambda _2 \ne 0$. If not, then we would get that

$$\begin{aligned} a\alpha ^{n}=\left( \dfrac{d_1\cdot 10^{\ell _1}-(d_1-d_2)}{9}\right) \cdot 10^{\ell _2}. \end{aligned}$$

As before, we apply the automorphism $\sigma$ of the Galois group ${\mathcal {G}}$ on both sides and take absolute values as follows.

$$\begin{aligned} \left| \left( \dfrac{d_1\cdot 10^{\ell _1}-(d_1-d_2)}{9}\right) \cdot 10^{\ell _2}\right| =\left| \sigma (a\alpha ^{n})\right| = \left| c\gamma ^{n}\right| <1, \end{aligned}$$

which is false. Thus, $\Lambda _2\ne 0$. So, we apply Theorem 3.1 on (4.8) with the data:

$$\begin{aligned}&t:=3, \quad \eta _1:=\dfrac{d_1\cdot 10^{\ell _1}-(d_1-d_2)}{9a}, \quad \eta _2:=\alpha , \quad \eta _3:=10, \quad b_1:=1, \\&\quad b_2:=-n, \quad b_3:=\ell _2. \end{aligned}$$

As before, we have that $\ell _2<n$. Thus, we can take $B:=n$. Similarly, ${\mathbb {Q}}(\eta _1, \eta _2, \eta _3)={\mathbb {Q}}(\alpha )$, so we take $D:=3$. Furthermore, we have

$$\begin{aligned} h(\eta _1)= & {} h\left( \dfrac{d_1\cdot 10^{\ell _1}-(d_1-d_2)}{9a}\right) \\\le & {} h(d_1\cdot 10^{\ell _1}-(d_1-d_2)) + h(9a)\\\le & {} h(d_1\cdot 10^{\ell _1})+h(d_1-d_2)+h(9)+h(a)+\log 2\\\le & {} h(d_1)+\ell _1 h(10)+ h(d_1)+h(d_2)+h(9)+h(a)+2\log 2\\\le & {} \ell _1\log 10+4\log 9+\dfrac{1}{3}\log 44+2\log 2\\\le & {} 1.96\cdot 10^{14}(1+\log n)+4\log 9+\dfrac{1}{3}\log 44+2\log 2 \quad (\hbox {by } (4.6))\\< & {} 1.98\cdot 10^{14}(1+\log n). \end{aligned}$$

Thus, we can take

$$\begin{aligned} A_1:=5.94\cdot 10^{14}(1+\log n), \quad A_2:=\log \alpha , \quad \hbox {and} \quad A_3:=3\log 10. \end{aligned}$$

Theorem 3.1 tells us that

$$\begin{aligned} \log |\Lambda _2|> & {} -1.4\cdot 30^{6}\cdot 3^{4.5}\cdot 3^2(1+\log 3)(1+\log n) (5.94\cdot 10^{14}(1+\log n))(\log \alpha )(3\log 10)\\> & {} -6.77\cdot 10^{27}(1+\log n)^2. \end{aligned}$$

Comparing the above inequality with (4.7) gives,

$$\begin{aligned} n\log \alpha - \log 2 < 6.77\cdot 10^{27}(1+\log n)^2, \end{aligned}$$

which is equivalent to

4.9 $$\begin{aligned} n < 2.24 \cdot 10^{28}(\log n)^2. \end{aligned}$$

Applying Lemma 3.2 on (4.9) with the data $r=2$, $H:=2.24 \cdot 10^{28}$, and $L:=n$, gives

$$\begin{aligned} n<1.22\cdot 10^{32}. \end{aligned}$$

Lemma 4.1 implies that

$$\begin{aligned} \ell _1+\ell _2 < 3.24\cdot 10^{31}. \end{aligned}$$

We have just proved the following lemma.

#### Lemma 4.2

All solutions to the Diophantine equation (4.1) satisfy

$$\begin{aligned} \ell _1+\ell _2< 3.24\cdot 10^{31} \quad \hbox {and} \quad n< 1.22\cdot 10^{32}. \end{aligned}$$

### Reducing the bounds

The bounds given in Lemma 4.2 are too large to carry out meaningful computation. Thus, we need to reduce them. To do so, we apply Lemma 3.1 as follows.

First, we return to (4.4) and put

$$\begin{aligned} \Gamma _1:=(\ell _1+\ell _2)\log 10-n\log \alpha - \log \left( \frac{9a}{d_1}\right) . \end{aligned}$$

The inequality (4.4) can be rewritten as

$$\begin{aligned} \left| e^{-\Gamma _1}-1\right| < \dfrac{28}{10^{\ell _1}}. \end{aligned}$$

Assume that $\ell _1 \ge 2$, then the right–hand side in the above inequality is at most $7/25 <1/2$. The inequality $|e^z-1|<w$ for real values of *z* and *w* implies that $z<2w$. Thus,

$$\begin{aligned} |\Gamma _1| < \dfrac{56}{10^{\ell _1}}, \end{aligned}$$

which implies that

$$\begin{aligned} \left| (\ell _1+\ell _2)\log 10-n\log \alpha - \log \left( \frac{9a}{d_1}\right) \right| < \dfrac{56}{10^{\ell _1}}. \end{aligned}$$

Dividing through by $\log \alpha$ gives

$$\begin{aligned} \left| (\ell _1+\ell _2)\frac{\log 10}{\log \alpha }-n+ \left( \frac{\log ({d_1}/{9a})}{\log \alpha }\right) \right| < \dfrac{56}{10^{\ell _1}\log \alpha }. \end{aligned}$$

So, we apply Lemma 3.1 with the data:

$$\begin{aligned} \tau :=\frac{\log 10}{\log \alpha }, \quad \mu (d_1):=\frac{\log ({d_1}/{9a})}{\log \alpha }, \quad A:=\dfrac{56}{\log \alpha }, \quad B:=10, \quad 1\le d_1\le 9. \end{aligned}$$

Let $\tau = [a_{0}; a_{1}, a_{2}, \cdots ]=[3; 1, 3, 1, 1, 14, 1, 3, 3, 6, 1, 13, 3, 4, 2, 1, 1, 2, 3, 3, 2, \cdots ]$ be the continued fraction expansion of $\tau$. We choose $M:=10^{32}$ which is the upper bound on $\ell _1+\ell _2$. With the help of Mathematica, we find out that the convergent

$$\begin{aligned} \dfrac{p}{q}= \dfrac{p_{62}}{q_{62}} = \dfrac{5067116767207083507605709005080661}{1341009632511071028566373818645201}, \end{aligned}$$

is such that $q=q_{62}>6M$. Furthermore, it yields $\varepsilon > 0.0893601$, and therefore either

$$\begin{aligned} \ell _1 \le \dfrac{\log \left( (56/\log \alpha ) q/\varepsilon \right) }{\log 10} < 35, \end{aligned}$$

Thus, we have that $\ell _1\le 35$.

For fixed $0\le d_2 < d_1\le 9$ and $1\le \ell _1 \le 35$, we return to (4.7) and put

$$\begin{aligned} \Gamma _2:=\ell _2\log 10 -n\log \alpha + \log \left( \dfrac{d_1\cdot 10^{\ell _1}-(d_1-d_2)}{9a}\right) . \end{aligned}$$

From the inequality (4.7), we have that

$$\begin{aligned} \left| e^{\Gamma _2}-1\right| <\dfrac{2}{\alpha ^{n}}. \end{aligned}$$

Since $n>200$, the right–hand side of the above inequality is less than 1/2. Thus, the above inequality implies that

$$\begin{aligned} \left| \Lambda _1\right| < \dfrac{4}{\alpha ^{n}}, \end{aligned}$$

which leads to

$$\begin{aligned} \left| \ell _2\log 10 -n\log \alpha + \log \left( \dfrac{d_1\cdot 10^{\ell _1}-(d_1-d_2)}{9a}\right) \right| <\dfrac{4}{\alpha ^{n}}. \end{aligned}$$

Dividing through by $\log \alpha$ gives,

$$\begin{aligned} \left| \ell _2\left( \frac{\log 10}{\log \alpha }\right) -n + \frac{\log \left( (d_1\cdot 10^{\ell _1}-(d_1-d_2))/9a\right) }{\log \alpha }\right| <\dfrac{4}{\alpha ^{n}\log \alpha }. \end{aligned}$$

Again, we apply Lemma 3.1 with the data:

$$\begin{aligned} \tau :=\dfrac{\log 10}{\log \alpha }, \quad \mu (d_1, d_2):=\frac{\log \left( (d_1\cdot 10^{\ell _1}-(d_1-d_2))/9a\right) }{\log \alpha }, \quad A:=\dfrac{4}{\log \alpha }, \quad B:=\alpha . \end{aligned}$$

We take the same $\tau$ and its convergent $p/q=p_{62}/q_{62}$ as before. We choose $\ell _2<10^{32}:=M$. With the help of Mathematica, we get that $\varepsilon > 0.0000798749$, and therefore

$$\begin{aligned} n \le \dfrac{\log \left( (4/\log \alpha ) q/\varepsilon \right) }{\log \alpha } < 143. \end{aligned}$$

Thus, we have that $n\le 143$, contradicting the assumption that $n>200$. Hence, Theorem 2.1 is proved.
